# Dataset for a full-year time series characterization of separately collected organic fraction of municipal solid waste from rural and urban regions in Germany

**DOI:** 10.1016/j.dib.2021.107543

**Published:** 2021-11-07

**Authors:** Gregor Sailer, Johanna Eichermüller, Jens Poetsch, Sebastian Paczkowski, Stefan Pelz, Hans Oechsner, Joachim Müller

**Affiliations:** a University of Applied Forest Sciences Rottenburg, Schadenweilerhof, Rottenburg 72108, Germany; b Faculty of Forest Sciences and Forest Ecology, Department of Forest Work Science and Engineering, University of Göttingen, Büsgenweg 4, Göttingen 37077, Germany; c State Institute of Agricultural Engineering and Bioenergy, University of Hohenheim, Garbenstrasse 9, Stuttgart 70599, Germany; d University of Hohenheim, Institute of Agricultural Engineering, Tropics and Subtropics Group, Garbenstrasse 9, Stuttgart 70599, Germany

**Keywords:** OFMSW, Waste management, Waste disposal, Waste characterization, Energy yield, Rural and urban bio-waste, Physico-chemical composition, Trace elements, Seasonal changes

## Abstract

In the municipal context and depending on the collection scheme, different waste streams are of relevance. This article contains year-round data on the chemical composition of organic fractions of municipal solid waste (OFMSW) of rural and urban origins. All samples were collected in the municipality of Tübingen, which is located in southern Germany. The sampling procedure was executed in accordance with standard procedures mentioned in the German Biowaste Ordinance. The data presented in this article include (1) sampling area and process specifications (2) organoleptic examinations (3) dry matter and organic dry matter contents (4) impurity concentrations and (5) elemental compositions (major, minor and trace elements). All datasets are presented as a time series for the year 2018. Thus, this article especially presents the influence of season and settlement structure on the physico-chemical characteristics of OFMSW. Researchers, waste management companies and municipalities can compare and expand their own OFMSW data with those presented in this article. The dataset can also be used to calculate energy yields of OFMSW when utilized in anaerobic digestion. Based on the data, it is also possible to discuss and to evaluate the material utilization of OFMSW-based digestates and compost products, especially with regard to concentrations of major, minor and trace elements. For further discussion, please refer to the original scientific article Sailer et al. (2021).

## Specifications Table

**Table:** 

Subject	Waste Management and Disposal
Specific subject area	Physico-chemical characterization and comparison of rural and urban organic fraction of municipal solid waste (OFMSW) samples for a one-year period.
Type of data	21 Tables and 7 Figures
How data were acquired	Datasets for rural and urban OFMSW were acquired using standard physico-chemical analyzes (methods in experimental design chapter) and instruments: • Fresh mass (FM) and dry matter (DM) contents through oven drying (UNP 700, Memmert, Schwabach, Germany) • Organic dry matter (oDM) contents with a muffle furnace (AAF 1100, Carbolite, Neuhausen, Germany) • C, H, N contents through elemental analyzer (vario MACRO cube, elementar, Langenselbold, Germany) • Trace elements (TE) through inductively coupled plasma-optical emission spectroscopy (ICP-OES) (Spectro Blue, ASX-260 auto sampler, SPECTRO Analytical Instruments, Kleve, Germany) The procedure for the organoleptic examination of OFMSW was based on sampling protocols following [2] and subjective assessments during the sampling process.
Data format	Raw, processed (mean values, aggregated) and analyzed data within this article; Excel spreadsheet in the Appendix for supplementary data on the sampling area, organoleptic examinations and ICP-OES analyzes
Parameters for data collection	Each sample collected in the course of the year 2018 was immediate processed (drying, sorting, crushing) and stored airtight in dry state until further experiments were carried out. Due to the year-round sample collection, physico-chemical analyzes were executed with the total number of samples in 2019. This procedure was chosen to optimize the sample handling. The whole amount of OFMSW was dried for the DM content determination of each sample. The repetition number varied between double and ninefold depending on the volume of the available drying vessels. The sorting analyzes and consequently the determination of impurity levels was done based on dry OFMSW samples. All impurities were excluded from all further chemical analyzes. Elemental compositions and oDM content analyzes were done in triplicate or quadruplicate for each sample. A suitable sampling process for OFMSW as a complex material with different ingredients and varying optical properties was a crucial factor for the data quality. A drum vehicle constantly mixed the total amount of OFMSW during the collection process. This procedure increased the homogeneity of the OFMSW amounts and facilitated the sampling process. However, detailed information on the sampling are and the established collection scheme can be found in the original research paper [1].
Description of data collection	An extensive and year-round dataset on the physico-chemical properties of rural and urban OFMSW. In Germany, OFMSW is a typical municipal solid waste that is collected separately via green or brown biowaste bins. The data collection includes the following parameters: organoleptic examination, DM contents, impurity concentrations, oDM contents and concentrations of 37 major, minor and trace elements (C, H, N, O, Al, Ag, As, B, Ba, Be, Bi, Ca, Cd, Co, Cr, Cu, Fe, K, Li, Mg, Mn, Mo, Na, Ni, Pb, Sb, Se, Sr, Ti, Tl, V, Zn, Ga, In, Si, P, S). In total, 42 samples (*n* = 22 for rural and *n* = 20 for urban OFMSW) were analyzed. Mean values and boxplot data for each elemental concentration are therefore based on 88 (rural) or 80 (urban) single measurements.
Data source location	All OFMSW samples were collected in the municipality of Tübingen, which is located in the state of Baden-Württemberg in southern Germany. Urban OFMSW samples originated from the inner city of Tübingen (*Kernstadt*), while rural OFMSW samples were collected in surrounding villages (*Kilchberg, Weilheim, Kreßbach, Bühl*). In the municipality of Tübingen, the OFMSW amount of each garbage truck collection was transported to the central collection site (*Schinderteich*), where the OFMSW was temporarily stored as a heap on a concrete surface. Therefore, the sampling was conducted at the central collection site (*Schinderteich*) of the Waste Disposal Association and Waste Management Corporation of the administrative district of Tübingen.
Data accessibility	Data are available in this article with additional data in Appendix
Related research article	Sailer et al. (2021), Characterization of the separately collected organic fraction of municipal solid waste (OFMSW) from rural and urban districts for a one-year period in Germany, Waste Management, Volume 131, July 2021, p. 471-482, https://doi.org/10.1016/j.wasman.2021.07.004

## Value of the Data

• For an efficient utilization of OFMSW, detailed data on physico-chemical properties, especially with regard to different settlement structures or seasonal changes are valuable. This article provides a year-round dataset for the characteristics of German OFMSW of rural and urban origins with constant sampling locations
• This data will be useful for biomass and bioenergy-researchers as well as for municipalities and waste management companies. OFMSW data can be used for the comparison or for identification of energetic and material use potentials
• OFMSW datasets are both relevant for the determination of practical application potentials (energetic and material utilization) and for the establishment of efficient biowaste value chains
• In the biogas or composting sector and from a legal perspective, this data may serve as a basis for discussion, particularly in relation to feedstock pre-treatment, technical process parameters and digestate or compost utilization

## 1 Data Description

**Fig. 1:**
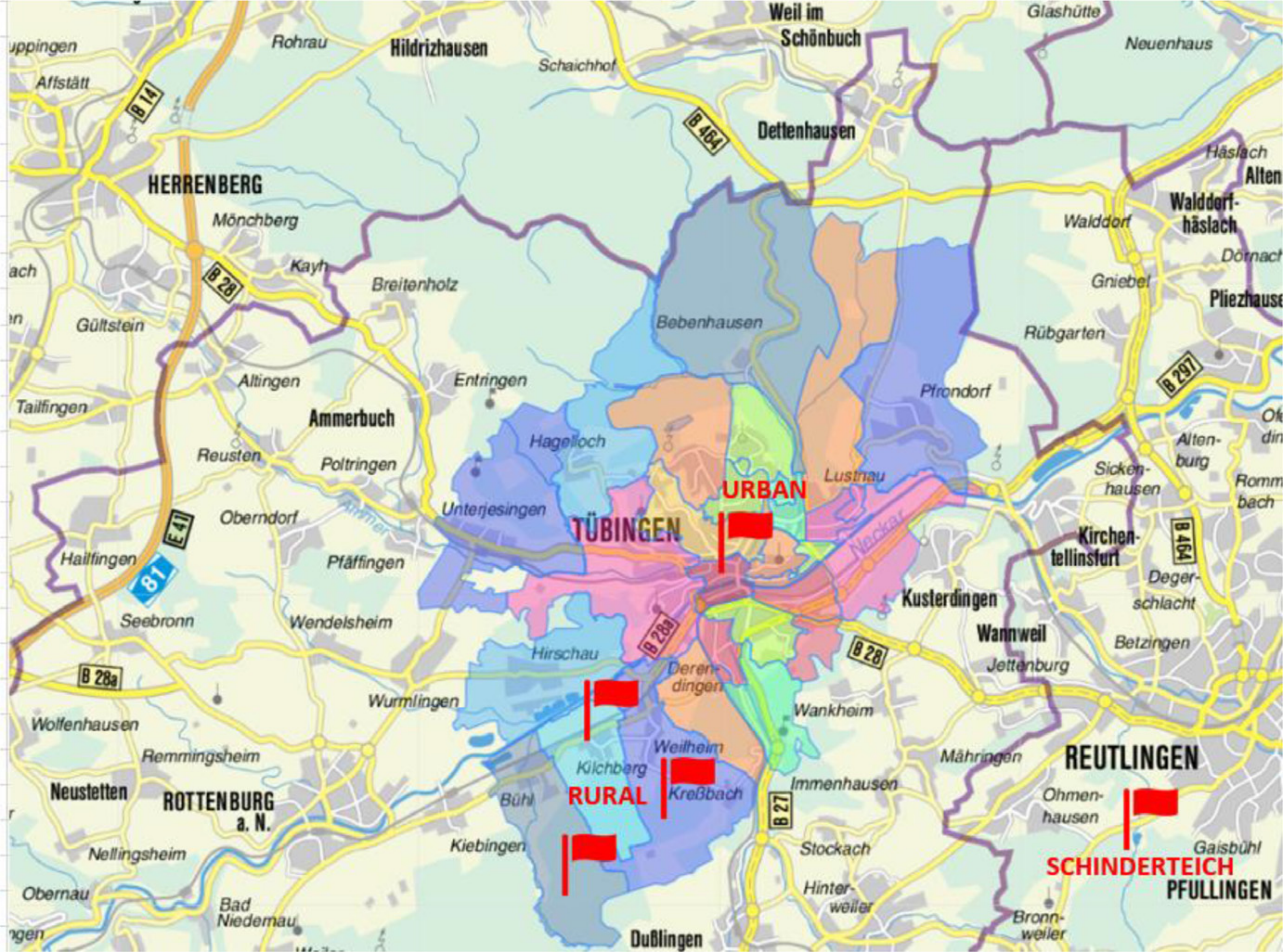
Municipality of Tübingen [9]. Sampling areas and the central collection site marked with flags.

**Fig. 2:**
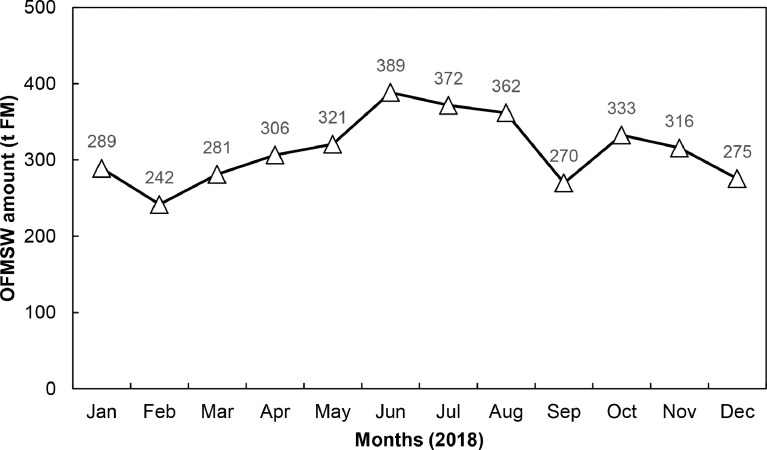
Total organic fraction of municipal solid waste (OFMSW) collection in tons fresh mass (t FM) for the year 2018 in the municipality of Tübingen (rural and urban areas) [11].

**Fig. 3:**
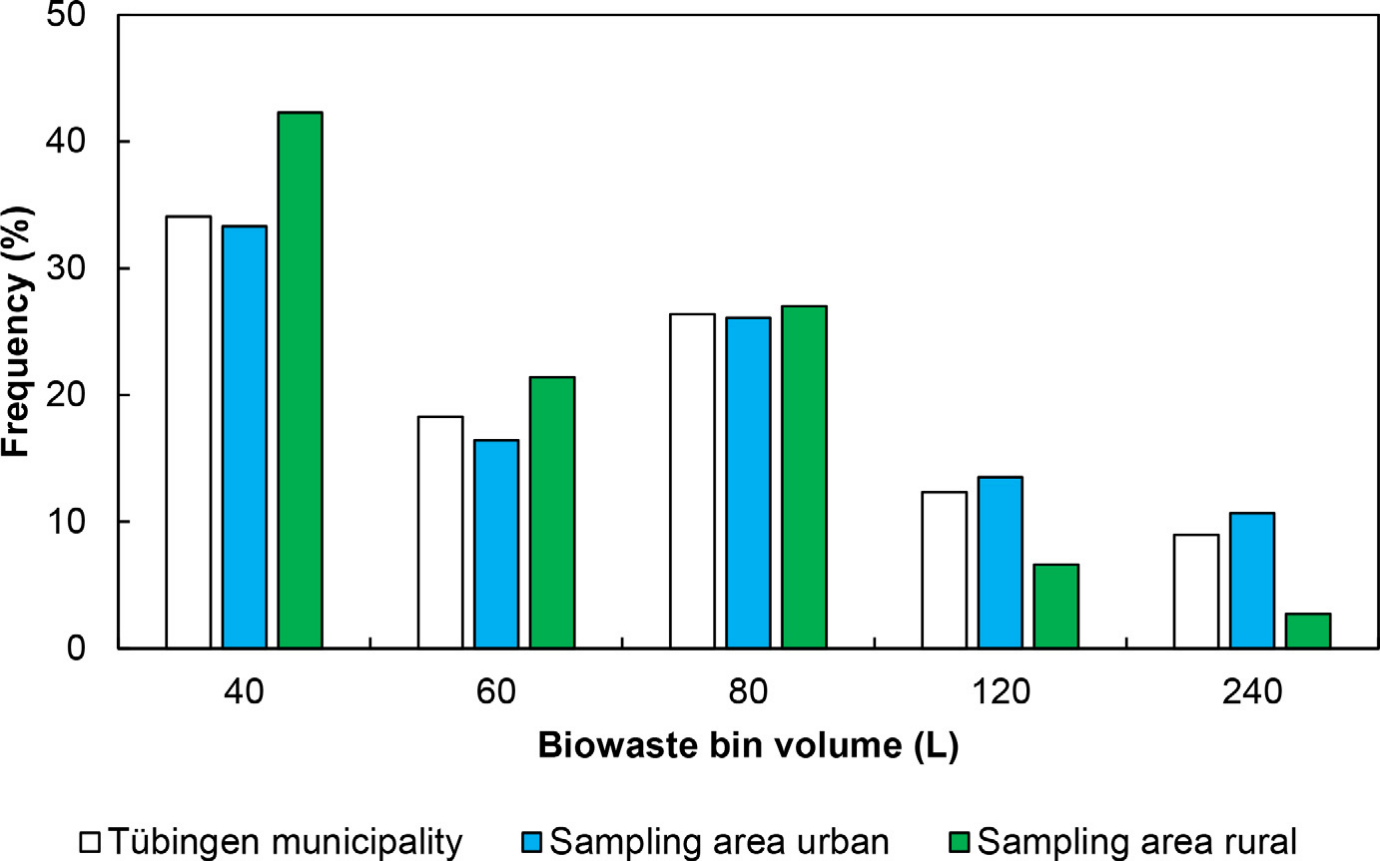
Size distribution of biowaste bins in the sampling areas compared to Tübingen municipality [11]. The raw data can be found in the Appendix A.

**Fig. 4:**
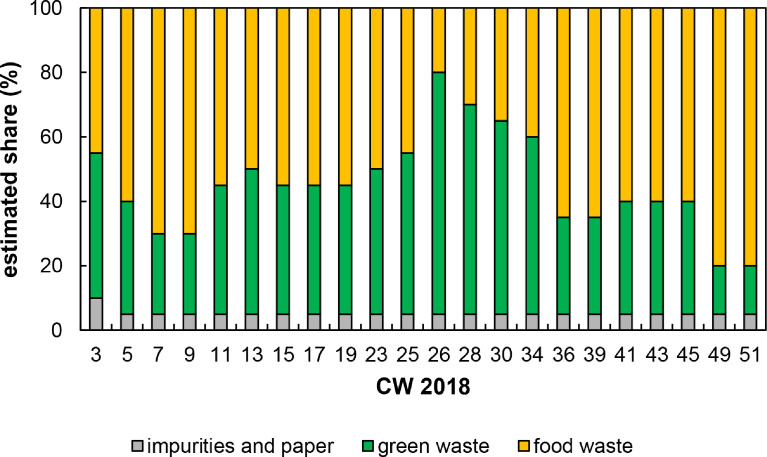
Estimated composition (determined during sampling process in each calendar week (CW)) of rural organic fraction of municipal solid waste (OFMSW) divided by the main waste types in the course of the year. The raw data can be found in the Appendix A.

**Fig. 5:**
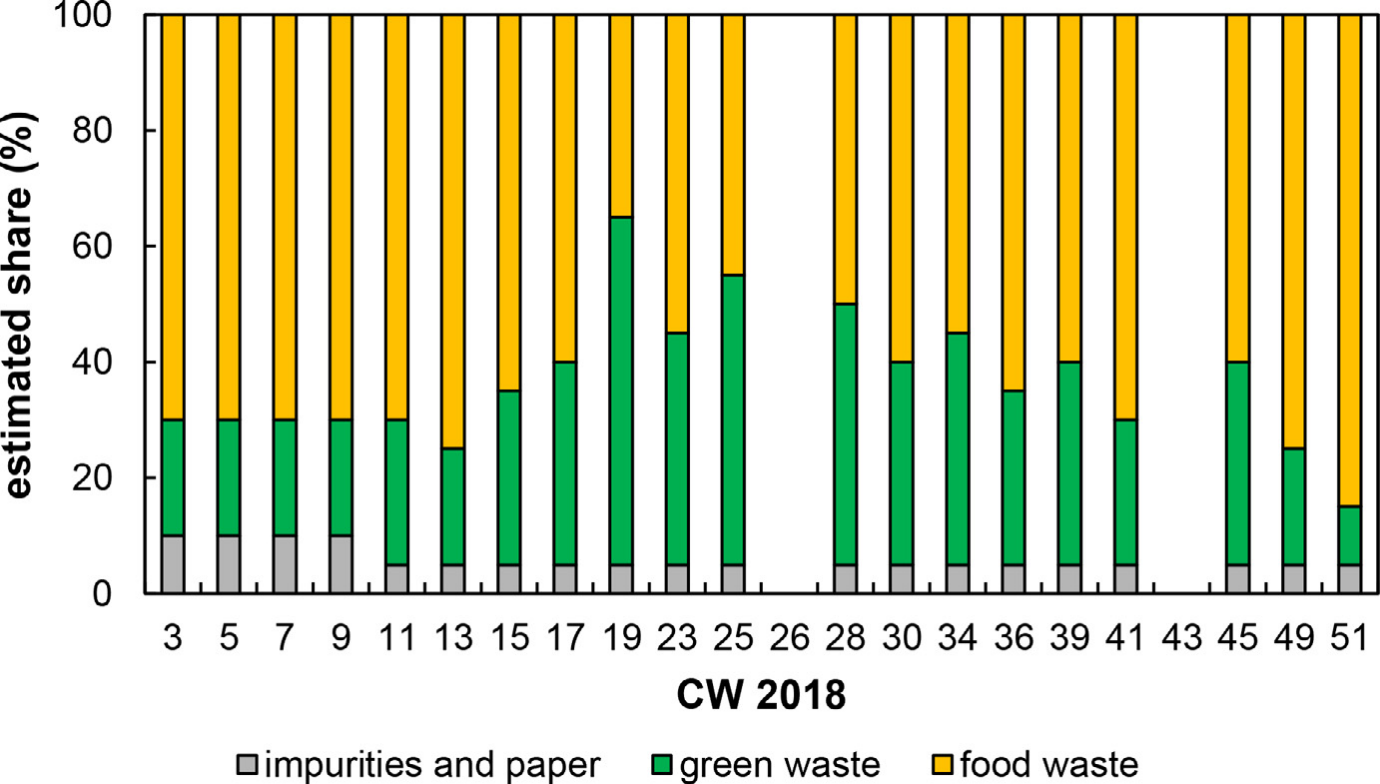
Estimated composition (determined during sampling process in each calendar week (CW)) of urban organic fraction of municipal solid waste (OFMSW) divided by the main waste types in the course of the year. The raw data can be found in the Appendix A.

**Fig. 6:**
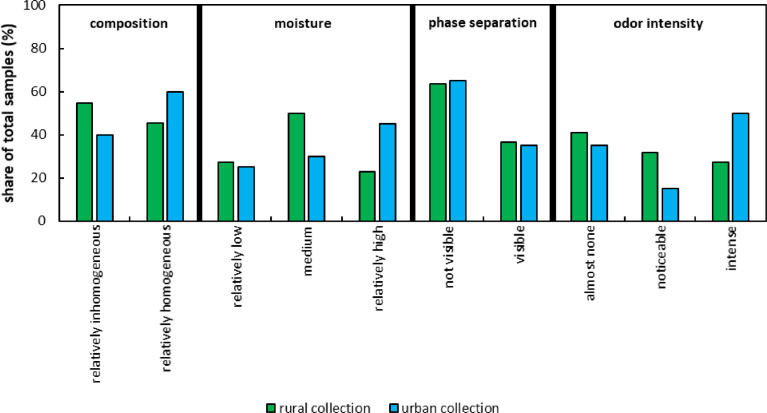
Organoleptic examination of rural and urban organic fraction of municipal solid waste (OFMSW) during the sampling process subdivided into the categories composition, moisture, phase separation and odor intensity. Composition indicates the level of overall homogeneity of the total OFMSW amount. Moisture describes the visual differences in terms of free or bound water in the sample (free water only occurred within the category relatively high). Phase separation depicts whether unloading processes of collection vehicles lead to partially higher shares of structural material (green waste). Odor intensity indicates the level of unpleasant smell from a distance of 5 m. The raw data can be found in the Appendix A.

**Fig. 7:**
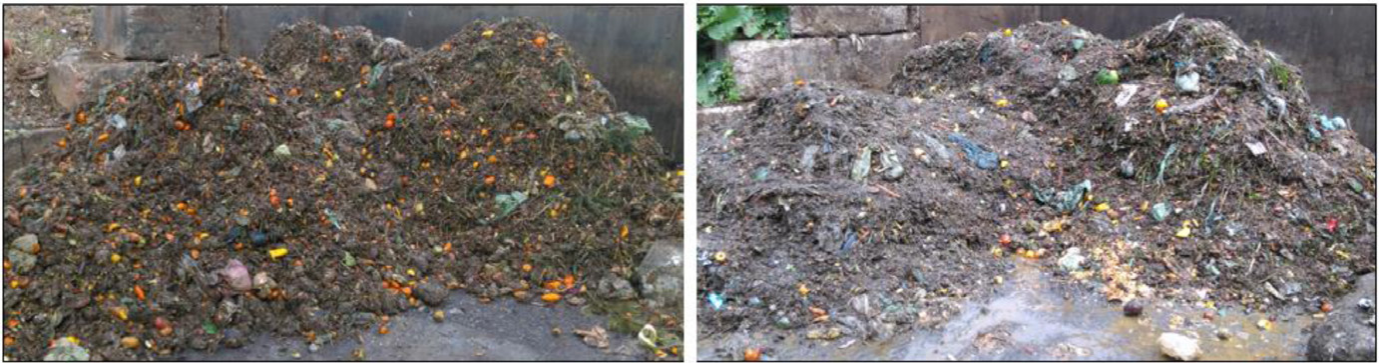
Rural organic fraction of municipal solid waste (OFMSW) in January (left) and in June (right) as an example for the organoleptic examination as described in Fig. 6.

Biomass is a common energy source and a versatile commodity with different characteristics depending on the origin or type. Available biomass types and their potential compositions as well as suitable conversion technologies have been reviewed within recent literature [3]. In addition to agricultural, silvicultural or aquatic biomass species, biogenic residues from municipalities such as the organic fraction of municipal solid waste (OFMSW) are available in large quantities. However, the characteristics of OFMSW depend on various parameters (e.g., season, collection scheme or geographical region). Within literature [4,5], several OFMSW characteristics have already been reviewed. In order to expand the existing literature, this Data in Brief (DIB) article provides a new dataset for the chemical composition of rural and urban OFMSW samples for a one-year period in Germany. All data are presented within this article - either in section two or in the form of supplementary data in the Appendix. Fig. 1 (municipality of Tübingen together with rural and urban sampling areas), Fig. 2 (amounts of OFMSW collected) and Fig. 3 (size distribution of biowaste bins) as well as Table 1 (general characterization of sampling areas) and Table 2 (number of biowaste bins in relevant areas) focus on the sampling areas with relevance for this article. Those data deliver background information and describe framework conditions that are relevant for the interpretation of all other data. Table 3 defines uniform sample codes used throughout the article and furthermore presents key facts of the sampling process for both rural and urban OFMSW. Table 4 and Fig. 4 (both relevant for rural OFMSW) as well as Table 5 and Fig. 5 (both relevant for urban OFMSW) show different sampling process data such as collection rate, total amount, sample amount, ambient temperature, density and estimated composition. Fig. 6 presents the analyzed data of the organoleptic examination during the sampling process itself. For a better understanding of Fig. 6 and all other data, Fig. 7 presents two typical OFMSW samples during different seasons. In addition, the raw data of Fig. 3, Fig. 4, Fig. 5–6 are also attached to the Appendix. Tables 6 and 7 (rural OFMSW) as well as Tables 8 and 9 (urban OFMSW) show single measurements and weighted mean values for dry matter (DM) contents in the course of the year. Further, Tables 10 and 11 present impurity levels (defined as sum of stones, metals, plastics) for rural and urban OFMSW samples. An isolated view on impurity categories such as stones or plastics is not available within this DIB article. Single measurements and mean values for organic dry matter (oDM) contents in rural OFMSW can be found in Tables 12 and 13 while oDM contents of urban OFMSW can be found in Tables 14 and 15. Single measurements and mean values for C, H and N contents of both OFMSW types are presented in Table 16, Table 17, Table 18, Table 19. Data for C, H and N can be used to estimate energy yield potentials based on fresh mass (FM), DM or oDM by calculating stoichiometric CH_4_ yields as described in the literature [6,7]. Therefore, values for S and O contents are needed additionally. S contents can be obtained by converting data of the inductively coupled plasma-optical emission spectroscopy (ICP-OES) to the designated reference unit (% DM). The calculation of O contents can be done by subtracting DM-based contents for C, H, N, S and ash from 100% DM. Tables 20 and 21 add information on boxplot data and mean values (annual values) of the ICP-OES analysis for rural and urban OFMSW. Single measurements and mean values for each sample and each major, minor or trace element (TE) can be found in the Appendix. The data presented can be used for the discussion of energy potentials as well as for the evaluation of OFMSW compositions especially with regard to legal limits that exist for impurities and TE such as heavy metals. In addition, conversion technologies such as the anaerobic digestion, delivering energy, digestates or compost products, can be assessed based on the data in this article.

**Table 1:** General characterization of sampling areas (the number of inhabitants relates to primary residence).

Sampling region	Area [10] (ha)	Inhabitants [9] and share of total inhabitants	Population density (inhabitants/km^2^)
**Tübingen municipality** (total)	10,812	87,579	810

**Sampling sites**			
**urban** (inner city)	108	6374 (7.3%)	5902
**rural** (Kilchberg, Weilheim, Kreßbach, Bühl)	1891	4759 (5.4%)	252

**Table 2:** Total number of biowaste bins in the city of Tübingen broken down into rural or urban sampling areas (31 December 2018) [11].

	Number of biowaste bins (-)		
Sampling region	Total	Households	Commercial
**Tübingen municipality**	9755	9258	497

**Sampling sites**			
**urban**	719*	674*	45*
**rural**	589	568	21

⁎ Calculated with data for the total city area of the municipality (6142 bins in total, 5754 households, 388 commercial) and the percentage of the urban sampling area (11.7%) based on the share of inhabitants in the urban sampling area (6300) compared to the total inhabitants of the city area (53,900) according to [10].

**Table 3:** Identification (ID) for each organic fraction of municipal solid waste (OFMSW) material and annotations referring to the sampling process.

Material	Remarks	ID
OFMSW Rural	• Separately collected; coarse impurities (stones, metals, plastics) manually removed before further processing and analytics • Sampling took place in calendar weeks (CW) 3, 5, 7, 9, 11, 13, 15, 17, 19, 23, 25, 26, 28, 30, 34, 36, 39, 41, 43, 45, 49 and 51 of the year 2018* • Numbers behind the ID only served as sample identifier • A press truck (due to repair measures of the drum vehicle) collected samples in CW 28, 30, 34, 51. All other samples were collected by drum vehicles • No sample was influenced by precipitation • Sampling was separated into seasonal types: CW 3-23 (winter/spring), 25-36 (summer) and 39-51 (autumn/winter) • The collection rate (weekly or biweekly) and therefore the maximum sample age depended on the season • Sampling took place between 9:30 a.m. and 1 p.m.	BT-KWKB

OFMSW Urban	• Separately collected; coarse impurities (stones, metals, plastics) manually removed before further processing and analytics • Sampling took place in CW 3, 5, 7, 9, 11, 13, 15, 17, 19, 23, 25, 28, 30, 34, 36, 39, 41, 45, 49 and 51 of the year 2018 (OFMSW in CW 26 and 43 not available)* • Numbers behind the ID only served as sample identifier • All samples were collected by drum vehicles • No sample was influenced by precipitation • Sampling was separated into seasonal types: CW 3-23 (winter/spring), 25-36 (summer) and 39-51 (autumn/winter) • The collection rate (weekly or biweekly) and therefore the maximum sample age depended on the season • Sampling took place between 9:30 a.m. and 1 p.m.	BT-TÜ

⁎ The sampling procedure had to be matched with the staff's schedule at the collection site. In some CW, the sampling of OFMSW was not possible (e.g., due to holidays). In CW 26 and 43, no urban OFMSW samples were available.

**Table 4:** Overview of the sampling process of rural organic fraction of municipal solid waste (OFMSW) for each calendar week (CW). The total amount of OFMSW refers to tons fresh mass (t FM).

CW	ID	Collection rate	Total amount (t FM)	Sample amount (g)	Ambient temp. (°C)	Density*(g/L)
3	BT-KWKB-1	biweekly	3.6	12,730	4	530
5	BT-KWKB-2	biweekly	8.4	9548	10	398
7	BT-KWKB-3	biweekly	8.0	10,308	2	429
9	BT-KWKB-4	biweekly	7.2	10,353	−10	575
11	BT-KWKB-5	biweekly	7.0	11,743	10	522
13	BT-KWKB-6	biweekly	9.2	8083	10	359
15	BT-KWKB-7	biweekly	9.5	9156	15	–
17	BT-KWKB-8	biweekly	10.0	9196	15	920
19	BT-KWKB-9	biweekly	9.1	5012	25	835
23	BT-KWKB-11	biweekly	9.5	5712	28	714
25	BT-KWKB-13	weekly	7.7	5539	25	1,108
26	BT-KWKB-14	weekly	1.4	3072	15	614
28	BT-KWKB-16	weekly	7.3	3035	30	303
30	BT-KWKB-18	weekly	7.3	3238	25	324
34	BT-KWKB-22	weekly	6.7	2904	30	323
36	BT-KWKB-24	weekly	6.0	5484	20	577
39	BT-KWKB-26	biweekly	9.8	4430	10	554
41	BT-KWKB-27	biweekly	9.8	3364	10	561
43	BT-KWKB-28	biweekly	9.4	3072	10	439
45	BT-KWKB-29	biweekly	10.0	3683	12	491
49	BT-KWKB-31	biweekly	9.3	4836	12	605
51	BT-KWKB-32	biweekly	8.5	3936	−4	394

⁎ Density values were calculated by dividing the sample FM with the sampling vessel volume (fill level was considered).

**Table 5:** Overview of the sampling process of urban organic fraction of municipal solid waste (OFMSW) for each calendar week (CW). The total amount of OFMSW refers to tons fresh mass (t FM).

CW	ID	Collection rate	Total amount (t FM)	Sample amount (g)	Ambient temp. (°C)	Density*(g/L)
3	BT-TÜ-1	biweekly	6.0	10,081	8	420
5	BT-TÜ-2	biweekly	7.0	7754	2	323
7	BT-TÜ-3	biweekly	6.4	11,755	−2	784
9	BT-TÜ-4	biweekly	5.1	9041	−8	335
11	BT-TÜ-5	biweekly	9.0	13,621	8	605
13	BT-TÜ-6	biweekly	6.1	6833	10	380
15	BT-TÜ-7	biweekly	4.2	4681	15	312
17	BT-TÜ-8	biweekly	8.0	9465	12	947
19	BT-TÜ-9	biweekly	2.0	4483	15	498
23	BT-TÜ-11	biweekly	7.7	7463	20	1,244
25	BT-TÜ-13	weekly	10.1	5170	25	1,034
26	–	weekly	–	–	–	–
28	BT-TÜ-16	weekly	10.3	4612	25	615
30	BT-TÜ-18	weekly	10.5	4953	25	550
34	BT-TÜ-22	weekly	6.3	4510	28	501
36	BT-TÜ-24	weekly	6.0	5552	25	617
39	BT-TÜ-26	biweekly	7.0	3245	20	649
41	BT-TÜ-27	biweekly	6.5	4400	15	629
43	–	biweekly	–	–	–	–
45	BT-TÜ-29	biweekly	7.6	4425	10	553
49	BT-TÜ-31	biweekly	unknown	3382	8	451
51	BT-TÜ-32	biweekly	12.5	5116	5	512

⁎ Density values were calculated by dividing the sample FM with the sampling vessel volume (fill level was considered).

**Table 6:** Fresh mass (FM) based dry matter (DM) contents of rural organic fraction of municipal solid waste (OFMSW) for each calendar week (CW). The sample size was reduced from nine to two in the course of the experiment.

CW	ID	DM (% m/m_FM_)								
3	BT-KWKB-1	36.54	38.77	35.87	37.53	35.07	34.25	33.88	38.38	35.73
5	BT-KWKB-2	30.73	31.73	32.16	35.68	31.65	30.25	31.58	–	–
7	BT-KWKB-3	31.90	35.06	32.06	32.82	34.82	41.72	–	–	–
9	BT-KWKB-4	34.09	29.20	29.02	35.50	33.39	30.78	–	–	–
11	BT-KWKB-5	35.80	36.69	35.33	34.32	35.40	32.07	–	–	–
13	BT-KWKB-6	33.24	32.87	34.06	34.74	33.63	40.69	–	–	–
15	BT-KWKB-7	33.82	35.00	34.95	35.64	34.46	35.37	36.75	–	–
17	BT-KWKB-8	39.76	36.27	35.83	35.20	–	–	–	–	–
19	BT-KWKB-9	34.38	33.69	34.43	34.15	34.12	33.68	–	–	–
23	BT-KWKB-11	29.16	30.17	–	–	–	–	–	–	–
25	BT-KWKB-13	30.77	30.03	–	–	–	–	–	–	–
26	BT-KWKB-14	28.20	33.83	–	–	–	–	–	–	–
28	BT-KWKB-16	26.31	29.04	–	–	–	–	–	–	–
30	BT-KWKB-18	33.08	33.55	–	–	–	–	–	–	–
34	BT-KWKB-22	32.82	38.15	–	–	–	–	–	–	–
36	BT-KWKB-24	30.49	33.29	–	–	–	–	–	–	–
39	BT-KWKB-26	30.59	30.41	–	–	–	–	–	–	–
41	BT-KWKB-27	32.44	32.41	–	–	–	–	–	–	–
43	BT-KWKB-28	34.06	33.20	–	–	–	–	–	–	–
45	BT-KWKB-29	32.97	32.11	–	–	–	–	–	–	–
49	BT-KWKB-31	33.02	33.79	–	–	–	–	–	–	–
51	BT-KWKB-32	29.36	30.33	–	–	–	–	–	–	–

**Table 7:** Mean values (weighted) and standard deviation (SD) for dry matter (DM) contents of rural organic fraction of municipal solid waste (OFMSW) for each calendar week (CW). All values are based on fresh mass (FM).

CW	ID	DM (% m/m_FM_) mean ± SD
3	BT-KWKB-1	36.31 ± 1.64
5	BT-KWKB-2	31.80 ± 1.63
7	BT-KWKB-3	35.01 ± 3.36
9	BT-KWKB-4	32.08 ± 2.48
11	BT-KWKB-5	34.95 ± 1.46
13	BT-KWKB-6	34.97 ± 2.67
15	BT-KWKB-7	34.91 ± 0.86
17	BT-KWKB-8	37.01 ± 1.77
19	BT-KWKB-9	34.07 ± 0.30
23	BT-KWKB-11	29.66 ± 0.51
25	BT-KWKB-13	30.40 ± 0.37
26	BT-KWKB-14	30.82 ± 2.81
28	BT-KWKB-16	27.72 ± 1.36
30	BT-KWKB-18	33.32 ± 0.24
34	BT-KWKB-22	35.56 ± 2.67
36	BT-KWKB-24	31.90 ± 1.40
39	BT-KWKB-26	30.51 ± 0.09
41	BT-KWKB-27	32.43 ± 0.02
43	BT-KWKB-28	33.64 ± 0.43
45	BT-KWKB-29	32.53 ± 0.43
49	BT-KWKB-31	33.41 ± 0.39
51	BT-KWKB-32	29.83 ± 0.49

**Table 8:** Single measurements for dry matter (DM) contents of urban organic fraction of municipal solid waste (OFMSW) for each calendar week (CW) based on fresh mass (FM). The sample size was reduced from six to two in the course of the experiment.

CW	ID	DM (% m/m_FM_)					
3	BT-TÜ-1	33.02	34.75	31.06	34.14	29.60	29.13
5	BT-TÜ-2	30.13	28.51	33.77	31.54	31.06	31.12
7	BT-TÜ-3	28.30	27.73	29.26	32.13	28.83	28.50
9	BT-TÜ-4	32.06	33.74	39.37	32.15	35.24	33.05
11	BT-TÜ-5	37.91	39.80	34.88	27.58	31.94	29.86
13	BT-TÜ-6	27.53	27.29	29.56	44.08	27.42	30.15
15	BT-TÜ-7	27.48	28.53	31.99	28.65	28.56	31.18
17	BT-TÜ-8	32.73	29.34	30.43	29.30	–	–
19	BT-TÜ-9	32.62	32.92	–	–	–	–
23	BT-TÜ-11	28.33	32.82	25.70	–	–	–
25	BT-TÜ-13	29.37	31.14	–	–	–	–
26	–	–	–	–	–	–	–
28	BT-TÜ-16	28.74	29.36	–	–	–	–
30	BT-TÜ-18	30.18	29.76	–	–	–	–
34	BT-TÜ-22	30.06	31.13	–	–	–	–
36	BT-TÜ-24	29.12	31.22	–	–	–	–
39	BT-TÜ-26	27.46	29.86	–	–	–	–
41	BT-TÜ-27	30.10	28.66	–	–	–	–
43	–	–	–	–	–	–	–
45	BT-TÜ-29	26.92	28.34	–	–	–	–
49	BT-TÜ-31	33.37	31.43	–	–	–	–
51	BT-TÜ-32	29.07	29.10	–	–	–	–

**Table 9:** Mean values (weighted) and standard deviation (SD) for dry matter (DM) contents of urban organic fraction of municipal solid waste (OFMSW) for each calendar week (CW). All values are based on fresh mass (FM).

CW	ID	DM (% m/m_FM_) mean ± SD
3	BT-TÜ-1	32.11 ± 2.16
5	BT-TÜ-2	30.98 ± 1.58
7	BT-TÜ-3	29.12 ± 1.42
9	BT-TÜ-4	34.49 ± 2.52
11	BT-TÜ-5	33.91 ± 4.31
13	BT-TÜ-6	30.09 ± 5.95
15	BT-TÜ-7	29.46 ± 1.61
17	BT-TÜ-8	30.48 ± 1.39
19	BT-TÜ-9	32.78 ± 0.15
23	BT-TÜ-11	29.11 ± 2.94
25	BT-TÜ-13	30.31 ± 0.88
26	–	–
28	BT-TÜ-16	29.05 ± 0.31
30	BT-TÜ-18	29.96 ± 0.21
34	BT-TÜ-22	30.57 ± 0.54
36	BT-TÜ-24	30.29 ± 1.05
39	BT-TÜ-26	28.67 ± 1.20
41	BT-TÜ-27	29.36 ± 0.72
43	–	–
45	BT-TÜ-29	27.67 ± 0.71
49	BT-TÜ-31	32.41 ± 0.97
51	BT-TÜ-32	29.08 ± 0.01

**Table 10:** Impurity concentrations (sum of all impurity types such as stones, metals, plastics) in rural organic fraction of municipal solid waste (OFMSW) based on dry matter (DM) and for each calendar week (CW).

CW	ID	Impurities (% m/m_DM_)
3	BT-KWKB-1	1.44
5	BT-KWKB-2	0.86
7	BT-KWKB-3	4.97
9	BT-KWKB-4	2.49
11	BT-KWKB-5	3.15
13	BT-KWKB-6	3.01
15	BT-KWKB-7	1.99
17	BT-KWKB-8	3.85
19	BT-KWKB-9	0.98
23	BT-KWKB-11	2.89
25	BT-KWKB-13	1.50
26	BT-KWKB-14	0.13
28	BT-KWKB-16	1.23
30	BT-KWKB-18	3.02
34	BT-KWKB-22	1.02
36	BT-KWKB-24	3.35
39	BT-KWKB-26	2.85
41	BT-KWKB-27	3.40
43	BT-KWKB-28	4.80
45	BT-KWKB-29	2.37
49	BT-KWKB-31	6.21
51	BT-KWKB-32	6.66

**Table 11:** Impurity concentrations (sum of all impurity types such as stones, metals, plastics) in urban organic fraction of municipal solid waste (OFMSW) based on dry matter (DM) and for each calendar week (CW).

CW	ID	Impurities (% m/m_DM_)
3	BT-TÜ-1	6.11
5	BT-TÜ-2	0.93
7	BT-TÜ-3	7.38
9	BT-TÜ-4	10.54
11	BT-TÜ-5	6.76
13	BT-TÜ-6	4.53
15	BT-TÜ-7	2.94
17	BT-TÜ-8	3.50
19	BT-TÜ-9	0.78
23	BT-TÜ-11	7.08
25	BT-TÜ-13	3.30
26	–	–
28	BT-TÜ-16	7.36
30	BT-TÜ-18	6.60
34	BT-TÜ-22	7.91
36	BT-TÜ-24	8.11
39	BT-TÜ-26	2.90
41	BT-TÜ-27	1.01
43	–	–
45	BT-TÜ-29	5.52
49	BT-TÜ-31	6.25
51	BT-TÜ-32	1.97

**Table 12:** Single measurements for organic dry matter (oDM) contents of rural organic fraction of municipal solid waste (OFMSW) for each calendar week (CW) based on dry matter (DM).

CW	ID	oDM (% m/m_DM_)		
3	BT-KWKB-1	84.8	83.9	83.2
5	BT-KWKB-2	84.6	86.1	85.5
7	BT-KWKB-3	85.1	85.5	84.8
9	BT-KWKB-4	87.0	87.1	87.2
11	BT-KWKB-5	82.4	81.9	80.5
13	BT-KWKB-6	84.0	83.9	83.2
15	BT-KWKB-7	75.0	75.5	74.9
17	BT-KWKB-8	68.1	69.3	70.1
19	BT-KWKB-9	74.5	76.7	76.8
23	BT-KWKB-11	78.5	79.0	79.1
25	BT-KWKB-13	83.1	81.9	79.1
26	BT-KWKB-14	86.6	86.9	86.8
28	BT-KWKB-16	81.6	82.5	83.0
30	BT-KWKB-18	85.8	88.0	81.6
34	BT-KWKB-22	84.7	84.6	84.7
36	BT-KWKB-24	81.7	78.0	80.8
39	BT-KWKB-26	84.6	83.4	85.0
41	BT-KWKB-27	80.2	83.4	82.9
43	BT-KWKB-28	83.0	82.7	82.6
45	BT-KWKB-29	82.3	85.7	83.9
49	BT-KWKB-31	85.9	87.5	83.7
51	BT-KWKB-32	82.1	84.7	83.9

**Table 13:** Mean values and standard deviation (SD) for organic dry matter (oDM) contents of rural organic fraction of municipal solid waste (OFMSW) for each calendar week (CW) based on dry matter (DM).

CW	ID	oDM (% m/m_DM_) mean ± SD
3	BT-KWKB-1	84.0 ± 0.65
5	BT-KWKB-2	85.4 ± 0.62
7	BT-KWKB-3	85.1 ± 0.29
9	BT-KWKB-4	87.1 ± 0.07
11	BT-KWKB-5	81.6 ± 0.80
13	BT-KWKB-6	83.7 ± 0.35
15	BT-KWKB-7	75.1 ± 0.26
17	BT-KWKB-8	69.2 ± 0.86
19	BT-KWKB-9	76.0 ± 1.07
23	BT-KWKB-11	78.9 ± 0.26
25	BT-KWKB-13	81.3 ± 1.69
26	BT-KWKB-14	86.8 ± 0.09
28	BT-KWKB-16	82.3 ± 0.57
30	BT-KWKB-18	85.1 ± 2.64
34	BT-KWKB-22	84.7 ± 0.07
36	BT-KWKB-24	80.2 ± 1.59
39	BT-KWKB-26	84.4 ± 0.67
41	BT-KWKB-27	82.2 ± 1.40
43	BT-KWKB-28	82.8 ± 0.16
45	BT-KWKB-29	83.9 ± 1.36
49	BT-KWKB-31	85.7 ± 1.57
51	BT-KWKB-32	83.6 ± 1.05

**Table 14:** Single measurements for organic dry matter (oDM) contents of urban organic fraction of municipal solid waste (OFMSW) for each calendar week (CW) based on dry matter (DM).

CW	ID	oDM (% m/m_DM_)		
3	BT-TÜ-1	90.3	90.2	90.6
5	BT-TÜ-2	87.4	87.3	87.5
7	BT-TÜ-3	88.9	89.0	88.5
9	BT-TÜ-4	88.3	88.6	89.3
11	BT-TÜ-5	86.2	85.5	86.2
13	BT-TÜ-6	87.7	88.3	87.8
15	BT-TÜ-7	81.0	81.7	82.1
17	BT-TÜ-8	82.5	82.1	84.4
19	BT-TÜ-9	72.3	73.0	73.0
23	BT-TÜ-11	83.9	83.9	84.7
25	BT-TÜ-13	80.7	80.1	79.6
26	–	–	–	–
28	BT-TÜ-16	80.2	81.9	82.4
30	BT-TÜ-18	83.9	84.7	77.6
34	BT-TÜ-22	83.4	85.0	84.6
36	BT-TÜ-24	86.5	85.8	86.7
39	BT-TÜ-26	83.5	84.5	84.9
41	BT-TÜ-27	83.0	83.8	81.0
43	–	–	–	–
45	BT-TÜ-29	88.6	89.1	86.1
49	BT-TÜ-31	84.4	discarded	85.7
51	BT-TÜ-32	87.4	87.6	87.0

**Table 15:** Mean values and standard deviation (SD) for organic dry matter (oDM) contents of urban organic fraction of municipal solid waste (OFMSW) for each calendar week (CW) based on dry matter (DM).

CW	ID	oDM (% m/m_DM_) mean ± SD
3	BT-TÜ-1	90.4 ± 0.17
5	BT-TÜ-2	87.4 ± 0.08
7	BT-TÜ-3	88.8 ± 0.25
9	BT-TÜ-4	88.8 ± 0.41
11	BT-TÜ-5	85.9 ± 0.31
13	BT-TÜ-6	87.9 ± 0.26
15	BT-TÜ-7	81.6 ± 0.46
17	BT-TÜ-8	83.0 ± 1.00
19	BT-TÜ-9	72.7 ± 0.32
23	BT-TÜ-11	84.1 ± 0.37
25	BT-TÜ-13	80.2 ± 0.46
26	–	–
28	BT-TÜ-16	81.5 ± 0.92
30	BT-TÜ-18	81.7 ± 3.17
34	BT-TÜ-22	84.3 ± 0.67
36	BT-TÜ-24	86.3 ± 0.40
39	BT-TÜ-26	84.3 ± 0.59
41	BT-TÜ-27	82.6 ± 1.19
43	–	–
45	BT-TÜ-29	87.9 ± 1.33
49	BT-TÜ-31	85.0 ± 0.66
51	BT-TÜ-32	87.4 ± 0.25

**Table 16:** Single measurements for N, C and H contents of rural organic fraction of municipal solid waste (OFMSW) for each calendar week (CW) based on dry matter (DM).

CW	ID	N (% m/m_DM_)	C (% m/m_DM_)	H (% m/m_DM_)
3	BT-KWKB-1	1.65	36.96	4.60
		1.65	39.34	5.12
		2.31	38.21	4.82
		1.68	39.68	5.19
5	BT-KWKB-2	2.22	46.21	6.06
		2.20	42.69	5.72
		2.15	44.88	6.09
		2.07	42.98	5.73
7	BT-KWKB-3	2.16	43.96	5.89
		2.13	44.28	5.95
		2.10	44.01	5.81
		2.08	43.65	5.88
9	BT-KWKB-4	2.03	44.25	6.09
		1.97	44.00	6.15
		1.92	43.68	6.08
		2.30	44.42	6.19
11	BT-KWKB-5	1.95	42.60	5.79
		2.01	41.81	5.75
		2.12	41.41	5.70
		2.25	41.45	5.72
13	BT-KWKB-6	2.07	42.20	5.47
		1.66	43.30	5.69
		1.57	42.29	5.54
		1.88	42.81	5.67
15	BT-KWKB-7	2.98	38.91	4.96
		2.13	40.49	5.21
		2.12	40.10	5.23
		2.15	35.99	4.68
17	BT-KWKB-8	1.91	36.58	4.69
		1.69	38.12	4.96
		1.79	37.10	4.89
		2.01	37.64	4.73
19	BT-KWKB-9	2.11	37.87	4.86
		2.00	38.71	5.12
		2.04	38.98	5.16
		2.00	39.50	5.12
23	BT-KWKB-11	2.33	41.82	5.41
		2.23	42.68	5.62
		2.21	41.96	5.55
		2.19	40.26	5.23
25	BT-KWKB-13	1.97	40.10	5.27
		1.88	41.45	5.55
		1.89	42.75	5.81
		1.81	42.50	5.72
26	BT-KWKB-14	2.14	43.52	5.85
		1.99	44.46	6.02
		1.94	44.27	6.10
		1.96	44.38	6.06
28	BT-KWKB-16	1.90	42.65	5.50
		1.88	43.50	5.73
		1.93	42.93	5.57
		1.95	44.54	5.80
30	BT-KWKB-18	1.91	43.93	5.90
		1.93	45.68	6.27
		1.96	45.27	6.12
		1.79	44.50	6.15
34	BT-KWKB-22	1.69	44.39	5.87
		1.54	44.47	6.12
		1.79	42.84	5.68
		1.65	43.03	5.76
36	BT-KWKB-24	2.03	43.87	5.56
		1.62	41.02	5.33
		1.82	42.20	5.51
		1.76	42.38	5.58
39	BT-KWKB-26	2.27	42.74	5.61
		1.23	44.57	5.65
		1.95	42.76	5.73
		1.92	43.03	5.71
41	BT-KWKB-27	1.89	42.41	5.55
		2.34	42.11	5.61
		2.04	44.40	5.93
		–	–	–
43	BT-KWKB-28	2.17	44.91	5.62
		2.06	44.44	5.70
		1.66	42.35	5.46
		1.98	44.82	5.69
45	BT-KWKB-29	1.91	42.44	5.54
		1.90	43.37	5.71
		1.88	44.58	5.89
		1.89	41.77	5.47
49	BT-KWKB-31	2.20	44.33	5.89
		1.88	44.65	6.08
		2.02	45.16	6.13
		1.93	44.06	5.97
51	BT-KWKB-32	2.85	44.21	5.89
		2.31	43.98	5.98
		2.10	39.90	5.34
		2.05	40.60	5.50

**Table 17:** Mean values and standard deviation (SD) for N, C and H contents of rural organic fraction of municipal solid waste (OFMSW) for each calendar week (CW) based on dry matter (DM).

CW	ID	N (% m/m_DM_) mean ± SD	C (% m/m_DM_) mean ± SD	H (% m/m_DM_) mean ± SD
3	BT-KWKB-1	1.82 ± 0.28	38.55 ± 1.07	4.93 ± 0.24
5	BT-KWKB-2	2.16 ± 0.06	44.19 ± 1.44	5.90 ± 0.17
7	BT-KWKB-3	2.12 ± 0.03	43.98 ± 0.22	5.88 ± 0.05
9	BT-KWKB-4	2.06 ± 0.15	44.09 ± 0.28	6.13 ± 0.04
11	BT-KWKB-5	2.08 ± 0.11	41.82 ± 0.48	5.74 ± 0.03
13	BT-KWKB-6	1.80 ± 0.19	42.65 ± 0.44	5.59 ± 0.09
15	BT-KWKB-7	2.35 ± 0.37	38.87 ± 1.76	5.02 ± 0.22
17	BT-KWKB-8	1.85 ± 0.12	37.36 ± 0.58	4.82 ± 0.11
19	BT-KWKB-9	2.04 ± 0.04	38.77 ± 0.59	5.07 ± 0.12
23	BT-KWKB-11	2.24 ± 0.05	41.68 ± 0.88	5.45 ± 0.15
25	BT-KWKB-13	1.89 ± 0.06	41.70 ± 1.04	5.59 ± 0.20
26	BT-KWKB-14	2.01 ± 0.08	44.16 ± 0.37	6.01 ± 0.10
28	BT-KWKB-16	1.92 ± 0.03	43.41 ± 0.72	5.65 ± 0.12
30	BT-KWKB-18	1.90 ± 0.06	44.85 ± 0.68	6.11 ± 0.14
34	BT-KWKB-22	1.67 ± 0.09	43.68 ± 0.75	5.86 ± 0.17
36	BT-KWKB-24	1.81 ± 0.15	42.37 ± 1.01	5.50 ± 0.10
39	BT-KWKB-26	1.84 ± 0.38	43.28 ± 0.76	5.67 ± 0.05
41	BT-KWKB-27	2.09 ± 0.19	42.97 ± 1.02	5.70 ± 0.16
43	BT-KWKB-28	1.97 ± 0.19	44.13 ± 1.04	5.62 ± 0.10
45	BT-KWKB-29	1.90 ± 0.01	43.04 ± 1.06	5.65 ± 0.16
49	BT-KWKB-31	2.01 ± 0.12	44.55 ± 0.41	6.02 ± 0.09
51	BT-KWKB-32	2.33 ± 0.32	42.17 ± 1.94	5.68 ± 0.27

**Table 18:** Single measurements for N, C and H contents of urban organic fraction of municipal solid waste (OFMSW) for each calendar week (CW) based on dry matter (DM).

CW	ID	N (% m/m_DM_)	C (% m/m_DM_)	H (% m/m_DM_)
3	BT-TÜ-1	2.74	46.69	6.25
		2.63	46.93	6.34
		2.35	47.18	6.43
		2.12	46.48	6.29
5	BT-TÜ-2	2.19	45.22	6.00
		2.05	44.80	6.01
		1.93	44.47	5.79
		1.95	43.40	5.79
7	BT-TÜ-3	2.12	45.54	6.03
		1.90	45.83	5.99
		2.10	45.14	5.98
		2.21	45.66	6.07
9	BT-TÜ-4	2.42	44.24	6.05
		1.92	45.40	6.25
		1.93	44.75	6.15
		2.01	44.66	6.23
11	BT-TÜ-5	2.18	45.18	6.00
		2.09	45.52	6.12
		2.06	45.47	6.12
		1.97	45.01	6.03
13	BT-TÜ-6	2.22	45.73	5.97
		2.11	45.22	5.94
		3.04	44.75	5.90
		3.12	45.50	5.99
15	BT-TÜ-7	1.87	44.64	5.70
		2.20	43.48	5.70
		2.13	43.45	5.73
		2.16	44.00	5.80
17	BT-TÜ-8	2.13	42.73	5.45
		2.18	42.05	5.48
		1.17	46.59	5.77
		2.26	42.39	5.51
19	BT-TÜ-9	2.16	36.33	4.67
		2.14	38.78	5.11
		2.21	38.73	5.11
		2.19	39.09	5.12
23	BT-TÜ-11	2.05	44.11	5.71
		1.91	45.80	5.91
		2.10	44.61	5.86
		2.02	44.60	5.86
25	BT-TÜ-13	2.12	41.35	5.45
		2.14	41.15	5.51
		2.18	42.65	5.76
		–	–	–
26	–	–	–	–
		–	–	–
		–	–	–
		–	–	–
28	BT-TÜ-16	2.20	44.37	5.79
		1.92	40.55	5.30
		2.14	43.45	5.74
		2.02	41.84	5.50
30	BT-TÜ-18	2.64	44.87	5.92
		1.98	46.69	6.16
		2.19	43.94	5.87
		2.21	44.36	5.95
34	BT-TÜ-22	2.19	42.66	5.48
		1.88	44.50	5.79
		2.08	45.17	5.91
		2.31	45.45	5.98
36	BT-TÜ-24	2.01	43.87	5.71
		1.87	43.53	5.65
		1.92	44.90	5.96
		1.83	42.48	5.63
39	BT-TÜ-26	2.32	45.44	6.03
		2.22	45.26	6.05
		2.12	46.66	6.13
		2.31	45.87	6.17
41	BT-TÜ-27	2.14	44.36	5.82
		2.04	45.16	6.04
		2.23	45.64	6.08
		2.02	45.66	6.11
43	–	–	–	–
		–	–	–
		–	–	–
		–	–	–
45	BT-TÜ-29	2.53	46.46	6.00
		3.13	45.82	6.07
		3.11	46.54	6.17
		2.45	47.43	6.32
49	BT-TÜ-31	1.84	43.58	5.79
		1.72	41.96	5.64
		1.76	43.16	5.78
		1.77	42.93	5.73
51	BT-TÜ-32	1.99	45.31	5.88
		2.14	46.44	6.21
		2.17	46.30	6.19
		2.10	45.15	6.02

**Table 19:** Mean values and standard deviation (SD) for N, C and H contents of urban organic fraction of municipal solid waste (OFMSW) for each calendar week (CW) based on dry matter (DM).

CW	ID	N (% m/m_DM_) mean ± SD	C (% m/m_DM_) mean ± SD	H (% m/m_DM_) mean ± SD
3	BT-TÜ-1	2.46 ± 0.24	46.82 ± 0.26	6.33 ± 0.07
5	BT-TÜ-2	2.03 ± 0.10	44.47 ± 0.67	5.90 ± 0.11
7	BT-TÜ-3	2.08 ± 0.11	45.54 ± 0.25	6.02 ± 0.04
9	BT-TÜ-4	2.07 ± 0.21	44.76 ± 0.42	6.17 ± 0.08
11	BT-TÜ-5	2.08 ± 0.08	45.30 ± 0.21	6.07 ± 0.05
13	BT-TÜ-6	2.62 ± 0.46	45.30 ± 0.37	5.95 ± 0.03
15	BT-TÜ-7	2.09 ± 0.13	43.89 ± 0.48	5.74 ± 0.04
17	BT-TÜ-8	1.94 ± 0.44	43.44 ± 1.83	5.55 ± 0.13
19	BT-TÜ-9	2.18 ± 0.03	38.23 ± 1.11	5.01 ± 0.19
23	BT-TÜ-11	2.02 ± 0.07	44.78 ± 0.62	5.83 ± 0.08
25	BT-TÜ-13	2.15 ± 0.02	41.72 ± 0.66	5.57 ± 0.14
26	–	–	–	–
28	BT-TÜ-16	2.07 ± 0.11	42.55 ± 1.47	5.58 ± 0.19
30	BT-TÜ-18	2.26 ± 0.24	44.97 ± 1.05	5.98 ± 0.11
34	BT-TÜ-22	2.12 ± 0.16	44.45 ± 1.09	5.79 ± 0.19
36	BT-TÜ-24	1.91 ± 0.07	43.70 ± 0.86	5.74 ± 0.13
39	BT-TÜ-26	2.24 ± 0.08	45.81 ± 0.54	6.10 ± 0.06
41	BT-TÜ-27	2.11 ± 0.08	45.21 ± 0.53	6.01 ± 0.11
43	–	–	–	–
45	BT-TÜ-29	2.81 ± 0.32	46.56 ± 0.57	6.14 ± 0.12
49	BT-TÜ-31	1.77 ± 0.04	42.91 ± 0.59	5.74 ± 0.06
51	BT-TÜ-32	2.10 ± 0.07	45.80 ± 0.57	6.07 ± 0.14

**Table 20:** Minimum (MIN), first quartile (FQ), median (MED), third quartile (TQ), maximum (MAX), mean value (MEAN) and standard deviation (SD) for different trace elements (TE) in rural and urban organic fraction of municipal solid waste (OFMSW) based on mean values of every single sample. All values are based on mg/kg dry matter. Data for single measurements and mean values of each OFMSW single sample and TE are attached to the Appendix A.

Parameter	Al rural	Al urban	Ag rural^1^	Ag urban^1^	As rural	As urban^1^	B rural^1^	B urban^1^	Ba rural	Ba urban	Be rural^1^	Be urban^1^
MIN	2029	1873	0.0167	0.0164	0.1647	0.0164	1.875	1.834	33.29	21.14	0.0165	0.0163
FQ	3198	2452	0.0173	0.0182	0.4194	0.1010	1.937	1.864	59.99	36.12	0.0169	0.0166
MED	4440	2969	0.0178	0.0699	0.8000	0.4102	2.070	1.912	66.93	40.61	0.0172	0.0169
TQ	5590	3612	0.0512	0.1883	1.034	0.6616	8.370	1.954	75.23	52.23	0.0176	0.0174
MAX	9401	7179	0.9229	0.5923	2.888	2.644	38.67	4.104	108.22	142.23	0.0187	0.0185
MEAN	4617	3281	0.1138	0.1463	0.9002	0.5184	7.517	2.021	68.67	49.69	0.0173	0.0170
SD	1736	1212	0.2347	0.1751	0.6381	0.5771	9.009	0.482	19.44	26.06	0.0005	0.0005

Parameter	Bi rural^1^	Bi urban^1^	Ca rural	Ca urban	Cd rural^1^	Cd urban^1^	Co rural	Co urban	Cr rural	Cr urban	Cu rural	Cu urban

MIN	0.0165	0.0163	20210	21257	0.0167	0.0163	0.2152	0.0700	9.573	5.39	9.813	9.737
FQ	0.0169	0.0166	24067	26049	0.0171	0.0166	0.8017	0.3150	15.00	12.65	11.39	12.84
MED	0.0172	0.0171	27365	32093	0.0174	0.0171	1.381	0.4969	18.15	14.67	14.37	14.54
TQ	0.0176	0.0174	29235	35687	0.0177	0.0174	1.656	0.7988	25.13	21.07	17.33	18.06
MAX	0.0187	0.7130	44240	53402	0.0821	0.1251	3.324	2.486	65.87	47.43	151.6	26.67
MEAN	0.0173	0.0585	28130	32540	0.0209	0.0247	1.390	0.6675	22.41	17.81	21.92	15.74
SD	0.0005	0.1530	5745	8916	0.0136	0.0252	0.7846	0.5708	12.68	8.936	29.61	3.930

Parameter	Fe rural	Fe urban	Kr ural	Ku rban	Li rural	Li urban	Mg rural	Mg urban	Mn rural	Mn urban	Mo rural	Mo urban^1^

MIN	1319	1148	9279	10214	3.041	1.680	2094	1567	72.84	48.32	0.5079	0.0165
FQ	2692	1604	10959	11158	4.471	2.945	2643	2144	126.3	73.11	0.6060	0.3469
MED	3403	2027	11507	15442	5.410	3.451	2958	2542	166.0	92.41	0.8739	0.5463
TQ	4338	2883	12760	17115	6.345	4.371	3291	2876	186.6	109.8	1.082	0.6846
MAX	7641	6428	16061	21902	11.01	9.849	4826	4588	417.9	235.1	1.472	1.126
MEAN	3577	2466	11906	14723	5.649	3.836	3031	2641	174.3	102.2	0.8619	0.5073
SD	1492	1297	1587	3510	1.757	1.667	610.7	676.4	75.58	45.24	0.2733	0.3270

1 at least one of the values was at the detection limit.

**Table 21:** Minimum (MIN), first quartile (FQ), median (MED), third quartile (TQ), maximum (MAX), mean value (MEAN) and standard deviation (SD) for different trace elements (TE) in rural and urban organic fraction of municipal solid waste (OFMSW) based on mean values of every single sample. All values are based on mg/kg dry matter. Data for single measurements and mean values of each OFMSW single sample and TE are attached to the Appendix A.

Parameter	Na rural	Na urban	Ni rural	Ni urban	Pb rural	Pb urban	Sb rural^1^	Sb urban^1^	Se rural^1^	Se urban^1^
MIN	2830	3650	3.563	2.384	1.875	1.206	0.0165	0.0163	0.0167	0.0163
FQ	3686	4167	6.631	5.137	4.494	4.621	0.0169	0.0166	0.0171	0.0166
MED	4711	4896	7.716	5.854	5.301	6.221	0.0172	0.0169	0.0174	0.0171
TQ	5285	5531	10.38	9.153	9.227	10.13	0.0176	0.0174	0.0177	0.0174
MAX	5685	7423	24.98	18.65	49.26	20.31	0.0187	0.0185	1.754	0.3166
MEAN	4516	4929	9.366	7.460	8.109	7.212	0.0173	0.0170	0.1138	0.0343
SD	868.2	905.1	4.851	3.860	9.406	4.416	0.0005	0.0005	0.3667	0.0656

Parameter	Sr rural	Sr urban	Ti rural	Ti urban	Tl rural^1^	Tl urban^1^	V rural	V urban	Zn rural	Zn urban

MIN	42.89	42.14	86.14	87.10	0.0167	0.0163	2.846	2.790	34.66	28.78
FQ	51.58	46.29	150.8	98.31	0.0171	0.0167	6.111	3.348	45.00	64.32
MED	54.23	50.39	223.3	112.9	0.0176	0.0173	7.879	4.412	55.74	152.6
TQ	67.50	54.08	269.8	141.5	0.0185	0.0176	9.422	5.790	77.39	224.2
MAX	77.76	91.31	396.4	292.2	0.1433	0.0286	17.32	14.12	646.9	368.3
MEAN	58.10	52.58	220.7	131.0	0.0335	0.0183	8.23	5.413	86.54	147.6
SD	10.08	10.36	90.84	52.98	0.0374	0.0031	3.488	2.892	123.5	88.54

Parameter	Ga rural	Ga urban^1^	In rural^1^	In urban^1^	Si rural	Si urban	P rural	P urban	S rural	S urban

MIN	4.712	4.683	2.974	2.515	3144	2721	2129	2081	1498	1535
FQ	6.820	4.800	5.349	3.711	4722	3807	2631	2668	1697	1822
MED	7.841	5.266	6.849	6.350	5365	5465	2754	3219	1769	2437
TQ	8.653	7.182	7.921	8.364	5572	6129	3731	3677	1875	2770
MAX	12.26	12.42	18.83	13.00	5916	7601	5697	7084	2078	3100
MEAN	7.889	6.350	7.027	6.386	5038	5096	3191	3479	1778	2339
SD	1.962	2.106	3.245	2.858	752.0	1374	965.8	1140	141.8	496.5

1 at least one of the values was at the detection limit.

## 2 Experimental Design, Materials and Methods

Detailed descriptions for all sampling procedures and experimental methods can be found in the original research paper [1]. This DIB article mainly focuses on additional information regarding the sampling areas.

### 2.1 Sampling material, area and procedure

Fig. 1 presents the municipality of Tübingen together with the rural and urban sampling areas located in the state of Baden-Württemberg in southern Germany. Separately collected OFMSW (biowaste bin) served as a sampling material for all analyzes. Throughout the year, the sampling locations did not change.

All sampling procedures were executed in accordance with the German Biowaste Ordinance and followed standard procedures [2,8]. Urban OFMSW samples originated from the inner city of Tübingen (*Kernstadt*), while rural OFMSW samples sourced from surrounding villages (*Kilchberg, Weilheim, Kreßbach, Bühl*). Each OFMSW collection in the municipality of Tübingen was temporarily stored on a concrete surface at the central collection site (*Schinderteich*) of the Waste Disposal Association (*Zweckverband Abfallverwertung Reutlingen/Tübingen, ZAV*) and Waste Management Corporation of the administrative district of Tübingen (*Abfallwirtschaftsbetrieb des Landkreises Tübingen*). Therefore, the sampling location for all samples was at *Schinderteich,* which is located approx. 20 km away from the inner city of Tübingen.

According to [10] and presented in Table 1, the total surface area combining rural and urban districts of Tübingen municipality (10,812 ha) can be subdivided into settlements and traffic areas (2455 ha), agriculture (2931 ha) and forests (5231 ha) . No detailed classification except for the total surface area was available for the urban sampling area. It can be assumed that the total surface area represents only residential and commercial settlements as well as traffic areas. The total rural sampling area (1891 ha) is characterized by high shares of agricultural and garden surfaces (561 ha) as well as forestry areas (979 ha) while the share of settlement areas can be described as relatively low (126 ha). In order to classify the chosen sampling areas (rural and urban), population densities were calculated (Table 1) before starting the experiments.

According to the Statistical Office of the state of Baden-Württemberg and Tübingen municipality [10], the number of inhabitants in the municipality of Tübingen was 87,579 in the year 2017 (Table 1). A growth to 91,655 inhabitants until the year 2020 has been monitored but as the way of data presenting changed from “primary residence” to “total residents”, the data of 2017 were used. Based on data of the Waste Disposal Association Tübingen, 3754 tons FM of OFMSW were collected in 2018 (Fig. 2). Additional information on waste collection processes in the municipality of Tübingen and in the sampling areas can be found in Table 2 and Fig. 3. Due to the growing population, the amount of available OFMSW will increase in the future.

### 2.2 Analytical methods

This chapter presents a summarizing overview on the analytical methods that were executed for the generation of the OFMSW data. Extensive descriptions of all analytical methods can be found in the original research paper [1].

The organoleptic examination was based on the procedure according to guidelines [2] supplemented by own subjective assessments during the sampling process. The DM content of each OFMSW sample was determined by drying the whole sampling material at 105°C in a drying oven (UNP 700, Memmert, Schwabach, Germany) for at least 24 h [12].

The impurity concentrations were determined based on the dry OFMSW samples. Therefore, all impurities (metals, plastics, stones) were manually removed from the dry samples and their weight was measured. Thus, the impurity concentrations as presented in this DIB article describe the total mass of all impurities (sum of plastics, metals, stones). The remaining DM was manually pre-crushed, partitioned by a sample divider and then sieved with mesh sizes of 63 and 45 mm (flat screening machine AS400 control, Retsch, Haan, Germany). Afterwards, the coarse fraction was shredded (AXT rapid 2200, Bosch, Gerlinen-Schillerhöhe, Germany), recombined with the fine fraction and then milled to particle sizes of approx. 1 mm with a customary mixer equipped with chrome steel blades (WMF Kult Pro 1400 W, WMF Group, Geislingen-Steige, Germany). This procedure has to be considered when evaluating the elemental concentrations, especially those of Fe, Ni and Cr.

The contents of oDM were determined via incineration in a muffle furnace (AAF 1100, Carbolite, Neuhausen, Germany) in accordance with standard procedures [13] by using approx. 1 g of DM in a ceramic crucible. Elemental analyzes (C, H, N contents) were carried out with an elemental analyzer (vario MACRO cube, elementar, Langsenbold, Germany) for all OFMSW samples [14]. Thereby, approx. 40 mg DM were pressed into a zinc foil coated tablet for each single measurement of each sample. S was not measured simultaneously in favour of the measurement accuracy for C, H and N. Instead, S and other TE were measured via ICP-OES [15] after digestion in aqua regia. Therefore, 300 mg DM of each sample were transferred into 50 mL Teflon vessels and combined with 1 mL of H_2_O_2_. Before microwave digestion at 190°C, 3 mL HNO_3_ (69%) and 9 mL HCL (35%) were added. The digested residues were aliquoted to 50 mL with aqua bidest and measured with the ICP-OES system (Spectro Blue, ASX-260 auto sampler, SPECTRO Analytical Instruments, Kleve, Germany). The solid residues consisting mainly of Si were separated by a centrifuge before spectroscopy and their mass was deducted from the sample mass. Thus, the values for Si only represent a partial amount of the total concentration since Si is not completely digestible with aqua regia. When evaluating ICP-OES measurements, all values below the detection limit (highlighted in each table of this article and in the supplementary data) were equated with this limit. Hence, some of those values might be slightly overestimated as the actual values could be even lower than the detection limit (0 < value < detection limit).

## Ethics Statement

The authors declare that they have followed the rules of scientific research and publishing. No conflict of interest exists in this study.

## CRediT authorship contribution statement

**Gregor Sailer:** Conceptualization, Methodology, Validation, Formal analysis, Investigation, Visualization, Writing – original draft, Writing – review & editing. **Johanna Eichermüller:** Conceptualization, Methodology, Validation, Investigation. **Jens Poetsch:** Conceptualization, Supervision. **Sebastian Paczkowski:** Conceptualization, Formal analysis, Writing – review & editing. **Stefan Pelz:** Conceptualization, Project administration, Funding acquisition, Writing – review & editing, Supervision. **Hans Oechsner:** Writing – review & editing, Supervision. **Joachim Müller:** Conceptualization, Methodology, Validation, Formal analysis, Visualization, Writing – original draft, Writing – review & editing, Supervision.

## Declaration of Competing Interest

The authors declare that they have no known competing financial interests or personal relationships, which have or could be perceived to have influenced the work reported in this article.

## Appendix Supplementary materials

**Appendix A:** Detailed data on ICP-OES analyzes and organoleptic examinations of each rural and urban OFMSW sample as well as the biowaste bin size distribution in the sampling area can be found in an additional file (Excel spreadsheet). Image, application 1
